# An analysis of spatial and temporal uncertainty propagation in agent-based models

**DOI:** 10.1098/rsta.2024.0229

**Published:** 2025-04-02

**Authors:** Yahya Gamal, Alison Heppenstall, William Strachan, Ricardo Colasanti, Kashif Zia

**Affiliations:** ^1^ Urban Big Data Centre, University of Glasgow School of Social and Political Sciences, Glasgow, UK; ^2^ The Alan Turing Institute, London, UK; ^3^ Social and Public Health Sciences Unit, University of Glasgow School of Health and Wellbeing, Glasgow, UK; ^4^ College of Medical, Veterinary and Life Sciences, University of Glasgow, Glasgow, UK; ^5^ School of Geography, University of Leeds, Leeds, UK

**Keywords:** spatio-temporal uncertainty, agent based model, smoking behaviours, uncertainty quantification, total Sobol index

## Abstract

Spatially explicit simulations of complex systems lead to inherent uncertainties in spatial outcomes. Visualizing the temporal propagation of spatial uncertainties is crucial to communicate the reliability of such models. However, the current Uncertainty Analyses (UAs) either consider spatial uncertainty at the end of model runs, or consider non-spatial uncertainties at different model states. To address this, we propose a Spatio-Temporal UA (ST-UA) approach to generate an uncertainty propagation index and visualize the temporal propagation of different uncertainty measures between two temporal model states. We select the total effects sensitivity measure (a Sobol index) for a sample application within the ST-UA approach. The application is the Tobacco Town ABM, a spatial model simulating smoking behaviours. We showcase the effect of the statistical distributions of wages and smoking rates on the propensity to buy cigarettes, which leads to the propagation of uncertainty in the number of purchased cigarettes by individuals. The findings highlight the usefulness of the ST-UA in (i) communicating the reliability of the spatial outcomes of the model; and (ii) guiding modellers towards the spatial areas with relatively high uncertainties at different temporal steps. This approach can be readily transferred to other application areas that are characterized with spatio-temporal uncertainty.

This article is part of the theme issue ‘Uncertainty quantification for healthcare and biological systems (Part 2)’.

## 1. Introduction

Simulation models are useful tools to explore and simulate the effect of human behaviours on individual and public health. These health systems simulations can be broadly categorized, based on their representation of human behaviours, into macro-scale simulations and micro-scale behavioural simulations. Macro-scale simulations represent behavioural patterns through a set of non-linear formulae which can be fitted to generate different outcome patterns. This implicitly accounts for different behaviours and is commonly applied in epidemiological models [1].

Micro-scale behavioural simulations explicitly represent the decision structures of individual actors (e.g. humans), rather than fitting non-linear equations to account for these behaviours. Agent-based models (ABMs) fall into this category where agents represent entities with behavioural structures that temporally interact with each other and with their environment [2]. ABMs have been applied to health-related applications including disease spread due to human behaviours [3,4] and human interactions with disease vectors [5]. The spatial environment is particularly relevant in such ABMs where the movement and proximity of agents are key factors impacting the model outcomes [6].

The outcomes of such behavioural models are subject to variance partly due to the complex nature of the simulated systems. Quantifying the uncertainty of outcomes is one of the recognized challenges, particularly for ABMs [7]. Studies addressing such challenges have drawn from the Uncertainty Analysis (UA) field [8]—which is applicable to various models and real-world data [9]. In ABMs, UA approaches were extended to distinguish between different sources of uncertainty while considering aggregate model outcomes. This involves uncertainty measures that focus on the temporal change in non-spatial ABM outcomes [10], and measures that can represent spatial uncertainty at a given ABM temporal state [11]. However, the representation of spatio-temporal uncertainties simultaneously in ABMs remains unexplored despite the importance of the geospatial component within many ABMs [7].

To address this limitation, we propose a Spatio-Temporal UA (ST-UA) approach to represent the temporal propagation of an uncertainty measure for each spatial unit under analysis. We extend on spatial UA approaches proposed for non-temporal models [12–14] to generate a transferable framework for representing spatial uncertainty across the established UA indicators for ABMs. The proposed framework produces visual representations of the temporal propagation of uncertainty that can (i) guide modellers to spatial areas with high propagation of uncertainties between different temporal steps; and (ii) communicate uncertainty outcomes to a non-expert audience. To showcase the ST-UA framework, we apply it to the Tobacco Town (TT) ABM, a smoking behaviours ABM that simulates the movement of smokers between houses, work locations and cigarette outlets.

The paper is structured as stated hereafter. Section 2 provides a background on UA in ABMs followed by a brief overview of the application of UA approaches in health systems models. Section 3 describes the proposed ST-UA approach and highlights the total effects sensitivity index as a UA indicator that is compatible with this approach. Section 4, briefly describes the TT ABM and highlights the experiments applied to showcase the ST-UA approach. Section 4c shows and discusses the visual results of the TT ABM following the ST-UA approach. Finally, §5 reflects on the ST-UA framework and highlights some potential extensions.

## 2. Background

### (a) Uncertainty Analysis

Uncertainty Analysis (UA) is a broad term that can describe a set of methods that fall under two categories: (i) Sensitivity Analysis (SA); and (ii) Uncertainty Quantification (UQ). First, SA focuses on identifying the influence of a model’s input on the uncertainty of its outcomes [15]. It can be categorized into Local Sensitivity Analyses (LSAs) and Global Sensitivity Analyses (GSAs) [8,16]. LSAs explore the effects of changes in inputs within limited ranges on the outcomes; they do not aim to explore all the numeric space of input variables [8]. They are useful in analysing how sensitive models are to minor modifications in inputs. The LSA approaches are commonly applied due to their simplicity [8]; however, they do not provide an accurate representation of the sensitivity of outcomes in non-linear models [17,18]. LSAs implicitly require assuming the independence of inputs, and this can lead to ignoring the uncertainty due to the interactions between such inputs (e.g. [19,20]). By contrast, GSAs explore all the possible values of a set of inputs to analyse their effect on the uncertainty of the outcomes. They can be systematically applied by sampling different combinations of the input parameters to account for how they interact with each other—for example, using a Monte Carlo sampling approach (e.g. [21]). This makes them suitable for application in non-linear models [8,17].

Both LSAs and GSAs can be implemented following different methods including derivative-based methods, elementary effect methods, regional methods, and variance-based methods. Derivative-based methods vary one input within a limited range making them an LSA method [8]. They are applied using a One factor At a Time (OAT) sampling approach which tracks the changes in outcomes due to incremental changes in one input parameter. It remains one of the more commonly applied approaches due to its simplicity [12,22], despite the limitation that it ignores interactions between inputs and model design uncertainties [12,23]. Elementary effect methods extend on derivative-based methods by exploring all the possible values of an input parameter. They still follow an OAT approach making them an LSA method that does not capture the interactions between the input parameters [8]. Regional methods define a numeric range for an output that is relevant for analysis based on the research problem and aims of the SA [24]. They use standard sampling methods to find the sets of inputs that lead to the relevant and irrelevant output ranges. The statistical distributions of the inputs in the two sets are compared to identify the contribution of each input in reaching the relevant output range. This makes them a GSA method as they can capture the interactions between the inputs in each analysed set. Variance-based methods investigate different components of the output variance in relation to different input samples to understand the model sensitivity. They include a multitude of methods, the most common of which is the Sobol method [25]. The Sobol method generates a first-order index (contribution of an input individually on the output variance), higher order indices (contribution of the interaction between two or more parameters on the output variance) and a total sensitivity index (contribution of an input and all of its interactions on the output variance). Other notable approaches are the sampling-partial rank correlation coefficient and the Shapley effects. The sampling-partial rank correlation coefficient is an indicator of the effect of an input on an output after removing the linear effects of all the other inputs [26]. The Shapley effect is based in game theory with a focus on the unequal value of collaborating players’ contributions towards an outcome [27]. It is applied as a GSA approach by considering the model inputs as the players’ with the unequal contribution to the variance of outcomes. Mathematically, it provides a total sensitivity index similar to Sobol, but normalized to the number of considered interactions (i.e. normalized to the highest order of the respective Sobol index) [28].

Second, UQ uses output data to identify the sources of uncertainty given a set of defined inputs, including empirical data and the model structure. Four sources of uncertainty can be highlighted for behavioural models simulating complex systems [10]: (i) parameter uncertainty (uncertainty due to the selected parameters to represent in the model and their values); (ii) model uncertainty (uncertainty due to the model design abstractions from the real mechanisms and the interactions between the model components); (iii) ensemble variance (uncertainty due to stochastic elements in the model); and (iv) empirical uncertainty (uncertainty due to limitations in accurately observing the real system). Some implementations explore such uncertainties at different time steps, for instance for calibration purposes (e.g. [10]). The spatial representation of these uncertainty measures depends on the type of the measured output. The outputs can either represent the aggregate state of the simulated system (e.g. [29,30]), or be measured at the scale of each spatial unit (e.g. [12–14]). Within spatial models, inverse UQ approaches applied on heterogeneous spatial outcomes are limited to bi-variate representations of the mean and standard deviation in the outcomes in each spatial unit [13,14]. These approaches are inspired from geostatistical methods incorporating means of spatial data points to estimate/predict further spatial indicators with associated standard deviation error maps [12,31].

SA and UQ approaches have also been considered simultaneously. This has been proposed in models with spatially heterogeneous outcomes within an integrated Uncertainty and Sensitivity Analysis (US-A) proposed by Ligmann-Zielinska *et al.* [12] and extended by Jankowski *et al.* [13] and Ligmann-Zielinska *et al.* [14] to include bi-variate mean and standard deviation visualization maps. The US-A approach conceptualizes uncertainty as the variance in the model outputs in each spatial unit given a set of selected inputs. For the SA, the US-A applies a quasi-random radial sampling approach [32] and uses a Sobol method [33]. It provides a first-order and total uncertainty index [25] per spatial unit representing the contribution of each input parameter separately on the outcome variance. This approach has only been applied on spatial multicriteria models [12,13] with no applications on behavioural individual-level simulation models. Its approach does not consider or visualize the temporal propagation of uncertainty from one time step to another, and it limits the definition of uncertainty to standard deviations in spatial outcomes. These gaps are addressed within the proposed ST-UA approach in this paper.

### (b) Simulation models and uncertainty: health systems

Simulation models represent abstractions of reality that can be used to understand complex systems such as health, climate, population and land use change. The complex nature of such models leads to the emergence of different forms of uncertainties in their outcomes. To showcase such models and their UA approaches, health systems are used as a central example in alignment with the scope of the application in this paper. Climate, population and land suitability models are then used to highlight spatial UA applications in simulation models.

Health systems are complex, and their models can include heterogeneous actors which interact with each other and with their environment affecting the state of the system [34]. These mathematical models can be broadly categorized into Systems Dynamics Models (SDMs) and behavioural models [35]. On the one hand, SDMs commonly use mathematical differential equations to represent the behaviour of a system as a whole (i.e. macroscale) (e.g. [36–38]). These models are well-established particularly in the domain of public health research [39]. On the other hand, behavioural models explicitly represent small-scale interactions, such as human behaviours, that have a collective effect on the simulated system. This includes agent-based models (ABMs) which incorporate interacting agents with defined behavioural structures [2,7]. These models have been increasingly used as exploratory and policy guiding tools in public health research (e.g. [40,41]).

ABMs and SDMs approach uncertainty similarly despite their structural differences, with the exception of model uncertainty. The relevant outcome in each domain of applications frames the focus of its SA and UQ studies. We showcase this through disease transmission models (one of the most applied mathematical models of health systems [42]) and tobacco control models (a stylized application of a tobacco control ABM is used in this paper, although there is very little published literature of UA ABM applications in tobacco control).

Disease transmission SDMs and ABMs simulate the spread of disease following a Susceptible, Infected and Recovered (SIR) approach [43]. The outcomes of these models are represented as infection number curves [43,44], and they are subject to uncertainties due to limited knowledge of the disease transmission rates and the human behaviours affecting such rates [44]. SA approaches are commonly used in such models [45]; for instance, Chitnis *et al*. [46] apply a GSA Latin hypercube sampling to calculate partial rank correlation coefficients in a Tuberculosis transmission model and Legrand *et al*. [47] apply an LSA derivative-based approach on a malaria spread model. UQ approaches are applied in these models to investigate stochastic uncertainty, parameter uncertainty and model uncertainty [44,48] (as described in §2a). The visualization and communication of these uncertainties is a focal point for these models [42,49], particularly those informing policies for controlling disease outbreaks. In these models, uncertainty is commonly visualized through fixed-time descriptive statistics [49]. This involves comparing the values of different infection number curves at each modelled time step to generate confidence intervals for the relevant outcomes per time step (e.g. [50,51]). Some approaches also make cross-comparisons between different infection number curves based on the stage of outcome curve (e.g. at peak infections), rather than comparing the same time step [49]. This implicitly focuses on communicating the uncertainties in the magnitude of the outcome, rather than its temporal occurrence in the model [42]. Models with a spatial representation of disease spread (e.g. [52,53]) have an extra layer of spatial uncertainty to communicate. However, these models do not attempt to visually represent the spatial uncertainty of disease spread in each simulated spatial unit.

Tobacco control mathematical models commonly apply SDM approaches [54,55], with some notable ABM applications [40,56]. The outcomes of these models broadly focus on the number of smokers and tobacco consumption. SA approaches are mostly applied within the context of empirical uncertainty. They use a derivative-based OAT approach to vary the inputs within their empirical statistical restrictions—for instance, Kim *et al.* [57] apply a probabilistic sensitivity analysis for the SimSmoke model by selecting samples from all the estimated inputs based on their statistical restrictions (means and standard deviations). Other models vary one parameter within the empirical ranges, instead of generating variations for multiple inputs simultaneously—for instance, Nemeth *et al.* [58] follow an OAT approach to quantify uncertainty in the return on investments in tobacco control policies. Inverse UQ is discussed within the context of validating the models (see [59], for a review). Error values are calculated and compared with real world data to estimate the accuracy of the models. For instance, Levy *et al.* [60] and Szklo *et al.* [61] apply a validation for the SimSmoke model based on the smoking attributable deaths in different contexts. All the stated models do not include an explicit representation of space. Accordingly, the spatial heterogeneity of the outputs and their measured uncertainty are not considered. Further, tobacco control models representing smokers and retailers in space do not extend to spatial uncertainty (e.g. Tobacco Town ABM [40,56]).

This general lack of focus on spatial uncertainties in the aforementioned health models stems from their research problems and aims. These are usually framed around aggregate indicators such as the overall number of infections without a particular interest in the spatial outcomes. Thereby, other domains which strictly tie the spatial aspect to the research aims extend the UAs to spatial uncertainties. This includes climate models, population models and land suitability models.

Climate models include simulations with spatially explicit outputs such as affected areas in case of floods or ice sheet sizes. It follows that the applications of UAs in these models are also spatially explicit. To exemplify, Slater *et al*. [62] apply an OAT LSA approach for the output spatial land cover changes due to floods, and Hebeler *et al*. [63] apply a UQ analyses focusing on empirical uncertainty due to limited data on ice sheets alongside a derivative-based LSA for a selected set of input parameters. Population models are another example with explicit spatial distributions of demographies. For instance, Sinha *et al*. [11] apply an UQ analysis comparing the simulated spatial populations to the observed ones. Land suitability models also extend to spatial uncertainty—mostly at administrative areas or neighbourhoods; applications include simulating groundwater contamination [23] and waste disposal [64]. However, none of these spatial UA applications investigate simultaneously the spatio-temporal propagation of uncertainties in the model outputs. To address this, we propose a Spatio-Temporal Uncertainty Analysis approach hereafter.

## 3. Spatio-Temporal Uncertainty Analysis approach

The proposed Spatio-Temporal Uncertainty Analysis (ST-UA) approach builds on Ligmann-Zielinska *et al.* [12,14] and Jankowski *et al*.’s [13] spatially explicit Uncertainty and Sensitivity Analysis (US-A). It provides a set of indices to represent the temporal propagation of a spatial uncertainty measure. This makes it flexible for incorporating SA measures (e.g. output variance within an OAT approach) and UQ measures (e.g. ensemble variance and errors compared with empirical evidence [10]). To showcase the ST-UA approach, we use a total effects sensitivity indicator based on the Sobol [25,33] method—as described in the US-A approach [12]. Hereafter, we discuss in detail the ST-UA uncertainty propagation indices and their incorporation with the total effects sensitivity Sobol index.

### (a) ST-UA uncertainty propagation indices

**Figure 1.**
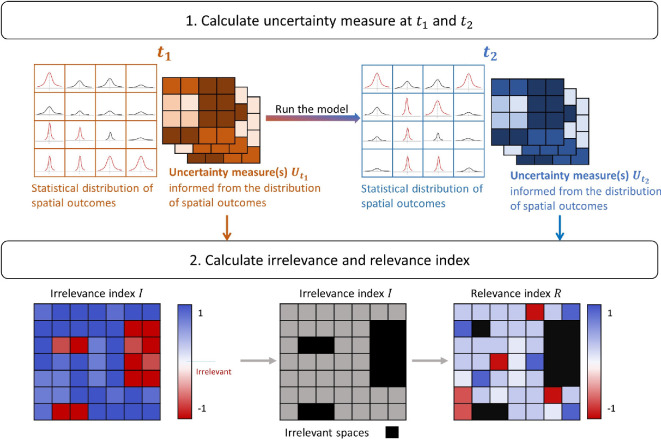
ST-UA schematic framework.

Applying the ST-UA requires initially identifying a spatial output measure ( p ) and two time steps of interest ( $t_{1}$ and $t_{2}$ ). For every output p , the following measures are defined at $t_{1}$ and $t_{2}$ : (i) an uncertainty irrelevance index; (ii) an uncertainty propagation relevance index; and (iii) the uncertainty measures of focus which can fall under SA or UQ approaches. A schematic representation of the ST-UA framework and the calculated indices is shown in figure 1.

The irrelevance index is generated to identify areas where the uncertainty measure at both $t_{1}$ and $t_{2}$ is relatively low, and therefore insignificant for further analysis. It is calculated as follows:


(3.1) $$I_{i,p|t_{1}\to t_{2}}=\alpha .(|U_{i,p|t_{1}}|+|U_{i,p|t_{2}}|)-\beta^{-(|U_{i,p|t_{1}}|+|U_{i,p|t_{2}}|)}\text{, where }\beta \geq 1$$

where $I_{i,p|t_{1}\to t_{2}}$ is the uncertainty propagation irrelevance index of an output p in a spatial unit i from time $t_{1}$ to $t_{2}$ , $U_{i,p|t_{1}}$ and $U_{i,p|t_{2}}$ are the uncertainty measures given times $t_{1}$ and $t_{2}$ , respectively, α is a factor influencing the irrelevance threshold and the increase in $I_{i,p|t_{1},t_{2}}$ due to the relevant values of $U_{i,p|t_{1}}$ or $U_{i,p|t_{2}}$ (electronic supplementary material, figure S1-A) and β is a factor influencing the irrelevance threshold and the convexity of the irrelevance function (electronic supplementary material, figure S1-B)—the condition β≥1 assures the function does not indicate high $U_{i,p|t_{1}}$ and $U_{i,p|t_{2}}$ cases as irrelevant. The irrelevance index informs the exclusion of spatial units with $I_{i,p|t_{1},t_{2}}\leq 0$ indicating that $U_{i,p|t_{1}}$ and $U_{i,p|t_{2}}$ are both insignificant for further analysis. We extend on the relation between the exclusion thresholds and the selected α and β values in §3b.

The uncertainty propagation relevance index from $t_{1}$ to $t_{2}$ is then calculated for each spatial unit $i^{{,}^{\prime }}$ satisfying the condition $I_{i^{\prime },p|t_{1},t_{2}}>0$ . The relevance index is used to identify the spatial units with a significant uncertainty measure at $t_{2}$ (high positive and low negative values) while differentiating between these spatial units based on the values of their uncertainty measure at $t_{1}$ . The relevance index is calculated as follows:


(3.2) $$R_{i^{\prime },p|t_{1}\to t_{2}}=\eta .(U_{i^{\prime },p|t_{2}}{)}^{3}+\rho .(U_{i^{\prime },p|t_{2}}.|U_{i^{\prime },p|t_{1}}|{)}^{3}$$

where $R_{i^{\prime },p|t_{1}\to t_{2}}$ is the uncertainty propagation relevance index of an output p in a spatial unit $i^{{,}^{\prime }}$ from the time $t_{1}$ to $t_{2}$ , η is a factor influencing the rate of increase of the relevance index with an increase in $U_{i^{\prime },p|t_{2}}$ only (electronic supplementary material, figure S2-A) and ρ is a factor influencing the relevance index at cases with high $U_{i^{\prime },p|t_{1}}$ and $U_{i^{\prime },p|t_{2}}$ values (electronic supplementary material, figure S2-B). The relevance index allows for categorizing the spatial units based on the relevance of their $U_{i,p|t_{1}}$ and $U_{i,p|t_{2}}$ as shown in table 1.

**Table 1.** Interpretation of relevance index values.

relevance index( R )	uncertainty index at $t_{1}$ ( $U_{t_{1}}$ )	uncertainty index at $t_{2}$ ( $U_{t_{2}}$ )
$R_{i^{\prime },p|t_{2}}<0$	relevant positive or negative	relevant negative
$R_{i^{\prime },p|t_{2}}>0$	relevant positive or negative	relevant positive
$R_{i^{\prime },p|t_{2}}\approx 0$	irrelevant	irrelevant

The range of $U_{i,p|t_{1}}$ and $U_{i,p|t_{2}}$ values considered as relevant is dependent on the selection of the factors α , β , η and ρ . This selection is a modeller’s decision and must be based on the $U_{i,p}$ values that indicate high uncertainty/sensitivity; this varies based on the type of uncertainty indicator and the tolerance to uncertainty in the simulated outcomes. We provide a generalized guideline for selecting such values hereafter, and we highlight brief OAT sensitivity analyses linking the values of the factors to the desired relevance and irrelevance thresholds where appropriate.

### (b) Selecting irrelevance and relevance index factors

The irrelevance index factors, α and β , dictate the uncertainty thresholds for including spatial units in the analysis. α controls the steepness of the irrelevance index $I_{i,p|t_{1}\to t_{2}}$ curve as shown in electronic supplementary material, figure S1-A in the appendix. This affects the threshold values of $U_{i,p|t_{1}}$ and $U_{i,p|t_{2}}$ that yield $I_{i,p|t_{1}\to t_{2}}\leq 0$ which are considered irrelevant. β affects the convexity of the $I_{i,p|t_{1}\to t_{2}}$ curve; it affects the rate of change of $I_{i,p|t_{1}\to t_{2}}$ with an increase of $U_{i,p|t_{1}}$ and $U_{i,p|t_{2}}$ as shown in electronic supplementary material, figure S1-B. Compared with α , β has a lower effect on the threshold values yielding irrelevant $I_{i,p|t_{1}\to t_{2}}$ values. Identifying such thresholds requires defining the value space of the uncertainty measures $U_{i,p|t_{1}}$ and $U_{i,p|t_{2}}$ corresponding to $I_{i,p|t_{1}\to t_{2}}>0$ indicating relevant cases. This value space can be defined using the following conditions:


(3.3) $$\left\{ \begin{array}{l} |U_{i,p|t_{1}}-U_{i,p|t_{2}}|>\lambda_{I}\\ |U_{i,p|t_{1}}+U_{i,p|t_{2}}|>\lambda_{I} \end{array}\right.$$

where $\lambda_{I}$ is a threshold defining the regions where the $U_{i,p|t_{1}}$ and $U_{i,p|t_{2}}$ values are indicated relevant for analysis. These conditions are accurate at β=1 , and they can be used as approximations if β>1 for simplicity.

The $\lambda_{I}$ value is a function of the α and β factors used to calculate $I_{i,p|t_{1}\to t_{2}}$ in equation (3.2). For clarity, table 2 provides an OAT SA for $\lambda_{I}$ at different α and β values. This SA can aid in the selection of the α and β values by following two steps: (i) identify the threshold $\lambda_{I}$ based on the tolerance to high propagation of uncertainty of outcomes—this is a modeller’s decision and depends on the selected uncertainty measure U ; (ii) select a combination of α and β values that generate the selected $\lambda_{I}$ (refer to table 2).

**Table 2.** Sensitivity of irrelevance threshold to α and β , bold text indicates the values used for exemplification in §3b.

α	1.0	1.2	1.4	1.6	1.8	2.0	2.2	2.4	2.6	2.8	3.0
$\lambda_{I}(\beta =1)$	1.00	0.83	0.71	0.62	0.56	0.50	0.45	0.42	0.38	0.36	0.33
$\lambda_{I}(\beta =1.2)$	0.86	0.73	0.64	0.56	0.51	0.46	0.42	0.39	0.36	0.34	0.31
$\lambda_{I}(\beta =1.4)$	0.77	0.67	0.59	0.52	0.47	0.43	0.40	0.37	0.34	0.32	0.30
$\lambda_{I}(\beta =1.6)$	0.71	0.62	0.55	0.50	0.45	0.41	0.38	0.35	0.33	0.31	0.29
$\lambda_{I}(\beta =1.8)$	0.67	0.59	0.52	0.47	0.43	**0.40**	0.37	0.34	0.32	0.30	0.28
$\lambda_{I}(\beta =2)$	0.64	0.56	0.50	0.46	0.42	0.38	0.36	0.33	0.31	0.29	0.28
$\lambda_{I}(\beta =2.2)$	0.62	0.54	0.49	0.44	**0.40**	0.37	0.35	0.32	0.30	0.29	0.27
$\lambda_{I}(\beta =2.4)$	0.59	0.53	0.47	0.43	0.39	0.36	0.34	0.32	0.30	0.28	0.26
$\lambda_{I}(\beta =2.6)$	0.58	0.51	0.46	0.42	0.38	0.36	0.33	0.31	0.29	0.27	0.26
$\lambda_{I}(\beta =2.8)$	0.56	0.50	0.45	0.41	0.38	0.35	0.33	0.30	0.29	0.27	0.26
$\lambda_{I}(\beta =3)$	0.55	0.49	0.44	0.40	0.37	0.34	0.32	0.30	0.28	0.27	0.25

To exemplify, consider a sensitivity index ranging from 0 to 1 indicting the fractional contribution of a parameter to the uncertainty of a model output. For a particular model, assume that the highest tolerated difference between the two indices from time step $t_{1}$ to $t_{2}$ is $\lambda_{I}=0.4$ (i.e. $|U_{i,p|t_{1}}-U_{i,p|t_{2}}|>0.4$ ). This means that the relevant regions include cases where the fractional contribution of the input on the uncertainty of the output increases by at least 0.4 (40%) from $t_{1}$ to $t_{2}$ . By referring to the $\lambda_{I}=0.4$ values in table 2, it can be identified that either α=1.8 and β=2.2 , or α=2 and β=1.8 are acceptable values.

The relevance index factors, η and ρ , affect the magnitude of relevance corresponding to the values of the uncertainty measures under analysis. High η values lead to steeper slopes for the relevance index ( $R_{i^{\prime },p|t_{1}\to t_{2}}$ ) function in relation to $U_{i,p|t_{2}}$ (electronic supplementary material, figure S2-A). This affects the $|U_{i,p|t_{2}}|$ values visualized as highly relevant. The key consideration to dictate η is to identify the threshold value of $\left(|U_{i,p|t_{2}}|\right)$ (labelled as $\left(\lambda_{U}\right)$ ) at which $|U_{i,p|t_{2}}|$ generates a high enough $R_{i^{\prime },p|t_{1}\to t_{2}}$ to be visually plotted. The choice of $\lambda_{U}$ and the threshold for visually emphasizing $R_{i^{\prime },p|t_{1}\to t_{2}}$ are modellers’ decisions, and they depend on the analysed uncertainty measure. For clarity, table 3 shows an OAT SA analysis of $\lambda_{U}$ to η at different $R_{i^{\prime },p|t_{1}\to t_{2}}$ values for visual emphasis (labelled as $\lambda_{R}$ )—assuming the analysed uncertainty measures range from 0 to 1. Accordingly, η can be selected by the following three steps: (i) identify the desired $\lambda_{U}$ value; (ii) identify the desired $\lambda_{R}$ value to be emphasized in the output visualizations; and (iii) refer to table 3 to identify the η value corresponding to the selected $\lambda_{R}$ and $\lambda_{U}$ values. To clarify, consider a case with a desired $\lambda_{U}=0.4$ and $\lambda_{R}=0.15$ for visual emphasis. By referring to (iii), this corresponds to η=2.4 at $\lambda_{R}=0.1$ .

**Table 3.** Sensitivity of $\lambda_{U}$ to η at different $\lambda_{R}$ values, bold text indicates the values used for exemplification in §3b.

η	1.0	1.2	1.4	1.6	1.8	2.0	2.2	2.4	2.6	2.8	3.0
$\lambda_{U}(\lambda_{R}=0.1)$	0.46	0.44	0.41	**0.40**	0.38	0.37	0.36	0.35	0.34	0.33	0.32
$\lambda_{U}(\lambda_{R}=0.15)$	0.53	0.50	0.47	0.45	0.44	0.42	0.41	**0.40**	0.39	0.38	0.37
$\lambda_{U}(\lambda_{R}=0.2)$	0.58	0.55	0.52	0.50	0.48	0.46	0.45	0.44	0.43	0.41	0.41
$\lambda_{U}(\lambda_{R}=0.25)$	0.63	0.59	0.56	0.54	0.52	0.50	0.48	0.47	0.46	0.45	0.44
$\lambda_{U}(\lambda_{R}=0.3)$	0.67	0.63	0.60	0.57	0.55	0.53	0.51	0.50	0.49	0.47	0.46

The ρ values affect the steepness of $R_{i^{\prime },p|t_{1}\to t_{2}}$ in simultaneously high $U_{i,p|t_{1}}$ and $U_{i,p|t_{2}}$ values (electronic supplementary material, figure S2-B). Higher ρ values emphasize such edge cases. To identify the $|U_{i,p|t_{1}}|$ and $|U_{i,p|t_{2}}|$ values affected by ρ , the following condition can be checked:


(3.4) $$\rho >{\left(\frac{1}{|U_{i,p|t_{1}}.U_{i,p|t_{2}}|}\right)}^{3}.$$

For instance, consider a case where $U_{i,p|t_{1}}=U_{i,p|t_{1}}=0.5$ is identified as an edge case. Solving equation (3.4) provides ρ>4 ; this is the lowest value of ρ that will emphasize the exemplified edge case.

The aforementioned $U_{i,p|t}$ values can represent different uncertainty measures, and accordingly the interpretation of what the relevance index values mean can vary. For consistency, we use a total effects sensitivity index [12] hereafter to showcase the ST-UA framework. This also highlights its applicability as an extension to the US-A approach [12–14].

### (c) Total effects sensitivity index

The total effects sensitivity index is introduced within the Sobol method that quantifies the effect of changes in inputs on the variance of outputs. Given a Monte Carlo sampling approach for the relevant model inputs, the total sensitivity index can be derived using two variance parameters: the variance V due to an input parameter j ; and the variance VC due all the inputs except j . First, the variance ( V ) is identified by running the model with the inputs generated within selected statistical distributions in each spatial unit i . This can be based on a modelling decision or on empirical statistical restrictions. In that empirical case, the sensitivity index can be conceptualized as the variance in an output due to j within the empirical boundaries of the simulated context. Second, the variance ( VC ) for each input j under analysis is measured by applying runs with controlled statistical distributions of j while sampling the rest using a quasi-random radial sampling approach [32]. The variance V due to all the input parameters is derived from the distribution of the results B , and the variance VC due to each parameter of interest separately is derived from the results $B_{k}^{*}$ —for an extended discussion on the sampling approach of the inputs to generate B and $B_{k}^{*}$ , see Appendix-(c) in electronic supplementary material. For each output of interest, V is calculated at the relevant temporal points as shown in equation (3.5), and VC is similarly calculated per input under analysis as shown in equation (3.6):


(3.5) Vk|i,t=∑k=1m(pk|i,t−Bk|i,t)m

(3.6) $$VC_{k|i,j,t}=\frac{\sum_{k=1}^{m}(p_{k|j}^{*}-{\bar{B^{*}}}_{k|i,j,t})}{m}$$

where $V_{k|i,t}$ is the variance in the k ^th^ output due to all the input parameters for the i ^th^ spatial unit at the t ^th^ time step in the model run, $VC_{k|i,j,t}$ is the variance due to the j ^th^ input parameter, $p_{k|i,t}$ is the value of the output, m is the total number of runs using different spatial input ensembles and Bk|i,t and B∗k|i,j,t are the means of the values in the result matrices generated in electronic supplementary material, equations S1 and S3, respectively (see Appendix-(c) in electronic supplementary material).

The V and VC values are then used to calculate a total effects sensitivity index $S_{i,j,p|t}$ as shown in equation (3.7) [12,25,33].


(3.7) $$\begin{array}{lcr} & S_{i,j,k|t}=\frac{V_{i,k|t}-VC_{j,k|t}}{V_{i,k|t}} & \end{array}$$

where $S_{j,p|t}$ is the total effects sensitivity index of the k ^th^ output in the i ^th^ spatial unit due to input j given time t , $V_{i,k|t}$ is the variance due to all the inputs and $VC_{j,k|t}$ is the variance due to all the inputs except for j . This sensitivity index is used as the uncertainty measure $U_{i,k|t}$ in equations (3.1) and (3.2). This makes the interpretation of the relevance index R in equation (3.2) subject to the meaning of the sensitivity index values as detailed in table 4.

**Table 4.** Interpretation of relevance index R given a total effects sensitivity measure is used.

relevance index	interpretation
$R_{i,j,k|t_{2}}<0$	significant uncertainty for an output p at $t_{1}$ and the inputs other than j significantly affect the output at $t_{2}$
$R_{i,j,k|t_{2}}>0$	significant uncertainty for an output p at $t_{1}$ and the input j significantly affect the output at $t_{2}$
$R_{i,j,k|t_{2}}\approx 0$	significant uncertainty for an output p at $t_{1}$ and insignificant uncertainty at $t_{2}$

This total effects sensitivity index can be interpreted as the fractional difference between the variance due to an input parameter j and the variance due to all the input parameters except j [12]. A positive $S_{i,j,k|t}$ value indicates that the output is more sensitive to j compared with all the parameters, whereas a negative $S_{i,j,k|t}$ value shows a higher sensitivity to all the parameters excluding j .

## 4. Application: Tobacco Town

To showcase the ST-UA approach, we use the Tobacco Town (TT) ABM for smoking behaviours which was previously applied in the context of California, USA [40]. The purpose of the model is to simulate smoking behaviours to inform spatial interventions (e.g. adding/removing cigarette outlets) that can impact public health. The model is compatible with the ST-UA approach because it includes (i) an explicit representation of spatial units (land plots); and (ii) output measures that vary across space. We show a brief description of the model following the template provided by Dilaver & Gilbert [65]. We provide details on the spatial structure of the model and the number of simulated agents in §4b. It should be noted that the application does not aim to provide an empirical model; instead, it provides a stylized model that is for spatial smoking behaviours with some inputs informed from California, USA. For a more detailed description of TT, refer to Luke *et al.* [40] and Hammond *et al.* [56]. The code of the model and the applied ST-UA analysis are also available in this GitHub repository (https://github.com/YahyaGamal/ST-UA_TobaccoTown).

### (a) Model description

**Figure 2.**
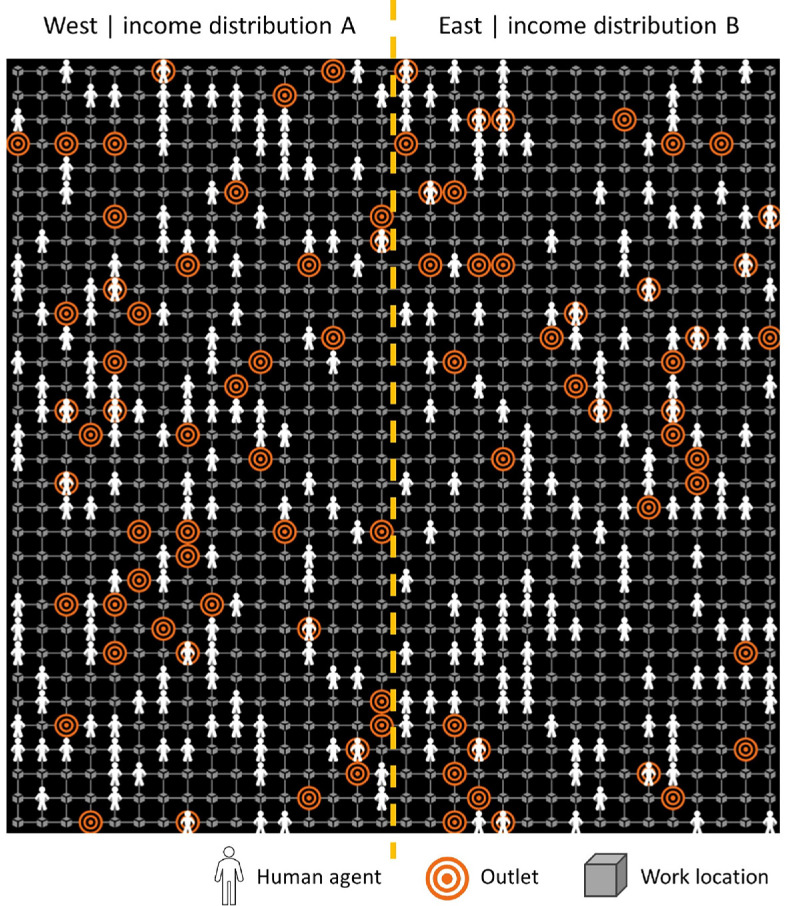
Sample spatial initialization of the TT ABM with human agents generated at their home location.

The model includes a set of agents that temporally interact with a set of objects in a virtual space. Each temporal step in the model represents a day, and the space is constructed as a grid of squared spatial units. Each spatial unit represents one land plot that can include multiple agents, but it can only include one object as shown in figure 2. Further details on the agents, their state variables and actions are described hereafter.

#### (i) Agents and objects

TT includes humans as the only type of agents. Humans are heterogeneous, and they can be either smokers or non-smokers. The ABM also includes two types of objects: (i) outlets; and (ii) work locations. Outlets represent retailers that can sell cigarette packs to humans. Work locations are areas that agents commute to each day. Each one of these objects take up one spatial unit only.

#### (ii) State variables

The agents have a set of state variables to dictate their home location, work location, smoking rate and income. The model also includes global variables that affect the choices of smokers. A list of the relevant state variables to the application in this paper is shown in table 5.

**Table 5.** State variables.

	state variable	description
human	home	the location which the agent lives in
	work	the work location at which the agent works
	home-work	the route between home and work
	nearest-outlet	the nearest outlet from the home-work route of the agent
	transport-type	the transport mode used to commute
	wage	the annual income of an agent
	inventory	the number of cigarette packs currently owned
	smoking-rate	the number of cigarettes smoked daily
	value-of-time	the value of time spent to purchase cigarettes (equation (4.1))
outlet	price	price of per cigarette pack excluding discounts
global	fuel-price	the price of fuel per mode of transport

#### (iii) Actions and interactions

Each day human agents execute a set of actions with the intention of achieving their work commitments and satisfying their smoking needs, if any. They execute two actions each time step: (i) commute between work and home; and (ii) visit a retailer to purchase cigarettes. First, human agents select a random path between work and home under the restriction that each spatial step must decrease the Euclidean distance between the agent and its target location. Human agents begin their time step (day) by moving towards their work. At the end of each day, they use the same path to commute back from work to home. Second, the agents visit the retailer in one of the two trips between home and work if they satisfy two conditions: (i) their smoking-rate is higher than zero; and (ii) the number of currently owned cigarettes (their inventory) is lower than their smoking-rate. At the retailer, the human agents calculate the prices of all the possible number of packets (each packet includes 20 cigarettes) they can purchase given their wage as shown in equation (4.1) [40]—noting that the price per packet varies based on the outlet and the number of purchased packets.


(4.1) $$C_{r(q)}=\left(\frac{d_{r}}{s}+\gamma \right).w.\zeta +d_{r}.\frac{f}{e}+q.C_{r(1)}$$

where $C_{r(q)}$ is the cost of purchasing a q amount of cigarette packs at retailer r , $d_{r}$ is the distance taken from the work-home route to reach the retailer, s is the travel speed which depends on the used mode of transport, w is the wage per year of the agent, γ is a factor representing the time in hours taken to make a cigarette purchase, ζ is the cost of time for a human agent, f is the fuel cost and e is the fuel efficiency and $C_{r(1)}$ is the price of purchasing one cigarette pack which is drawn from the outlet state variable ‘price’ in table 5.

The human agents select the number of packs q that yields the lowest price per cigarette pack. They make the purchases accordingly and resume their work-home trips.

### (b) Experiments

The introduced experiments aim to highlight the outcomes and visualizations of the ST-UA approach using the described TT ABM. The design of the experiments requires a clear definition of the following: (i) the spatial structure of the ABM; (ii) the state variables of the human agents including the variable of interest ( k in electronic supplementary material, equation S3); (iii) the observed outcome; (iv) the number of time steps to define the temporal points of interest; and (v) the number of run times needed to generate the required results matrices for a total effects sensitivity index ST-UA analysis ( B and $B_{k}^{*}$ in electronic supplementary material, equations S1 and S3).

First, the spatial structure of the model is initialized using a 32 × 32 grid of spatial units. An abstract space is used instead of the geographical space representing California, USA [40] in the interest of clear visualizations. The density of the human agents population, the work locations and the outlets are generated as per the urban rich areas in California, USA [40]. This leads to the spatial distribution of approximately 302 human agents, 1024 work places and 85 outlets as shown in figure 2. This spatial structure is consistently used at the initialization of all the runs of the experiments for comparability.

Second, for the state variables, we follow a partially controlled approach. That is, one state variable values are tightly controlled within defined statistical distributions and the rest remains within the empirical distributions of California, USA. Although this distances the model inputs from the accurate empirical distributions, it allows for showcasing the ST-UA framework in cases of high and low sensitivity—which is the aim of this application. We introduce two experiments: (i) the wages experiment; and (ii) the smoking rate experiment. In the wages (smoking rate) experiment, the initial values of the wages (smoking rate) of human agents are tightly controlled within defined normal distributions, whereas the rest of the state variables are generated as per the context of California, USA [40]. We subdivide the spacial context into western and eastern areas (figure 2), each with a different distribution of wages or smoking rates as shown in table 6. The western areas have wider normal distributions for the input under analysis in both experiments. Thereby, the variance in spatial outcomes in the east and the west are expected to differ in case the output considered is highly affected by different distributions of wages and smoking rates. This makes it useful to apply the ST-UA analysis using a total effects sensitivity index that considers variance due to the tightly controlled input wages or smoking rate state variable.

**Table 6.** Mean ( μ ) and standard deviations ( σ ) of input parameters in the east and west spatial areas at initialization.

experiment	parameter	μ	$\sigma_{west}$	$\sigma_{east}$
wages exp.	wages	50 000	5000	500
smoking rates exp.	smoking rates	20	10	2

Third, the observed outcome of interest is the mean number of purchased cigarettes by the human agents residing in each spatial unit. This makes the outcome spatially heterogeneous, which allows for different representations of the irrelevance index I and uncertainty propagation relevance index R in each spatial unit. This outcome is labelled as the mean spatial purchase hereafter.

Fourth, the number of time steps is restricted to 30 days. This allows each human agent to make at least one cigarette packet purchase in case it is a smoker. The two temporal points of interest selected are day 1 and day 30 so that we can compare between the mean spatial purchase values at the initial and final states of the model.

Finally, the number of simulations required is dictated within to two sets of runs, namely set ${,}_{V}$ and set ${,}_{VC}$ . Set set ${,}_{V}$ and set ${,}_{VC}$ are required to separately calculate V and VC values, respectively. The results of each must assure that the variance in the V or VC is statistically stable; this requires 3000 runs per set. In terms of inputs and outputs, set ${,}_{V}$ requires an input matrix A with 3000 samples for each state variable, and it generates a 3000 sample result matrix B which is then used to calculate V . The statistical distributions of all the state variables in A are drawn from empirical distributions representing California, USA. The only exception is that the wages or smoking which are drawn from the normal distributions in table 6. For the wages (smoking rates) experiments, set ${,}_{VC}$ requires an input matrix $A_{wages}^{*}$ ( $A_{rates}^{*}$ ) which is exactly similar to A except for the wages (smoking rates) column. In $A_{wages}^{*}$ ( $A_{rates}^{*}$ ), the wages (smoking rates) column is regenerated to create a different sample from that in A that still falls within the normal distributions in table 6. This generates a different output matrix $B_{wages}^{*}$ ( $B_{rates}^{*}$ ) that can be used to calculate the VC . For clarity, the input and output matrices in the context of the wages experiment can be formalized as follows:


(4.2) $$\begin{array}{r} A=\left[\begin{array}{cccccc} J_{1,inventory} & ... & J_{1,wages} & J_{1,rates} & ... & J_{1,n}\\ J_{2,inventory} & ... & J_{2,wages} & J_{2,rates} & ... & J_{2,n}\\ ... & & \\ J_{3000,inventory} & ... & J_{3000,wages} & J_{3000,rates} & ... & J_{3000,n} \end{array}\right]⟹B=\left[\begin{array}{c} P_{1}\\ P_{2}\\ ...\\ P_{3000} \end{array}\right] \end{array}$$

(4.3) $$\begin{array}{lcr} & \begin{array}{r} A_{wages}^{*}=\left[\begin{array}{ccccc} J_{1,inventory} & ... & J_{1,wages}^{*} & ... & J_{1,n}\\ J_{2,inventory} & ... & J_{2,wages}^{*} & ... & J_{2,n}\\ ... & & & & \\ J_{m,inventory} & ... & J_{m,wages}^{*} & ... & J_{3000,n} \end{array}\right]⟹B_{wages}^{*}=\left[\begin{array}{c} P_{1|wages}^{*}\\ P_{2|wages}^{*}\\ ...\\ P_{3000|wages}^{*} \end{array}\right] \end{array} & \end{array}$$

(4.4) $$\begin{array}{lcr} & \begin{array}{r} A_{rates}^{*}=\left[\begin{array}{ccccc} J_{1,inventory} & ... & J_{1,rates}^{*} & ... & J_{1,n}\\ J_{2,inventory} & ... & J_{2,rates}^{*} & ... & J_{2,n}\\ ... & & & & \\ J_{m,inventory} & ... & J_{m,rates}^{*} & ... & J_{3000,n} \end{array}\right]⟹B_{rates}^{*}=\left[\begin{array}{c} P_{1|rates}^{*}\\ P_{2|rates}^{*}\\ ...\\ P_{3000|rates}^{*} \end{array}\right] \end{array} & \end{array}$$

where equation (4.2) reflects the inputs and outputs of the runs of set*_V_* and equations (4.3) and (4.4) reflect the inputs and outputs of the runs of set ${,}_{VC}$ in the wages and smoking rates experiments, respectively. Each outcome element P∈B represents the outcome values in all the spatial units; i.e. P can be described as a matrix of outputs $p_{xy}$ across the spatial grid of the model. Therefore, the variance of outcomes included in the sets B , $B_{wages}^{*}$ and $B_{rates}^{*}$ can be calculated for each spatial unit i with the coordinates xy to represent $V_{i}$ , $VC_{i,wages}$ and $VC_{i,rates}$ .

Identifying such variance measures allows for generating the following visual outcomes per experiment: (i) the variance V map at day 1; (ii) the variance VC map at day 30; (iii) the variance ( VC ) map at day 1; (iv) the variance ( VC ) map at day 30; (v) the total effects sensitivity index U at day 1; (vi) the total effects sensitivity index U at day 30; (vii) the irrelevance index I map from day 1 to day 30; and (vii) the uncertainty propagation relevance index R map from day 1 to day 30. The results are further described and discussed hereafter.

### (c) Results

**Figure 3.**
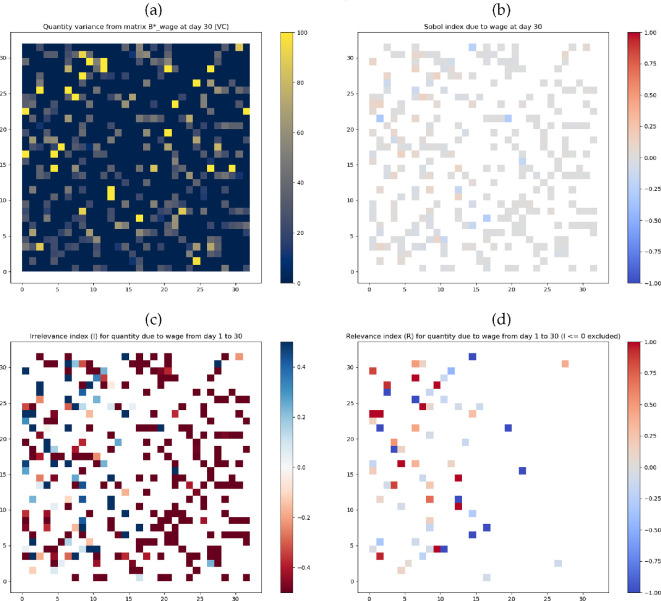
Wages experiment ST-UA outcomes: (*a*) $VC_{wages|t=30}$ ; (*b*) total effects sensitivity index $U_{wages|t=30}$ ; (*c*) $I_{wages}$ where α=2 and β=2 ; and (*d*) $R_{wages}$ where η=2 and ρ=1 .

**Figure 4.**
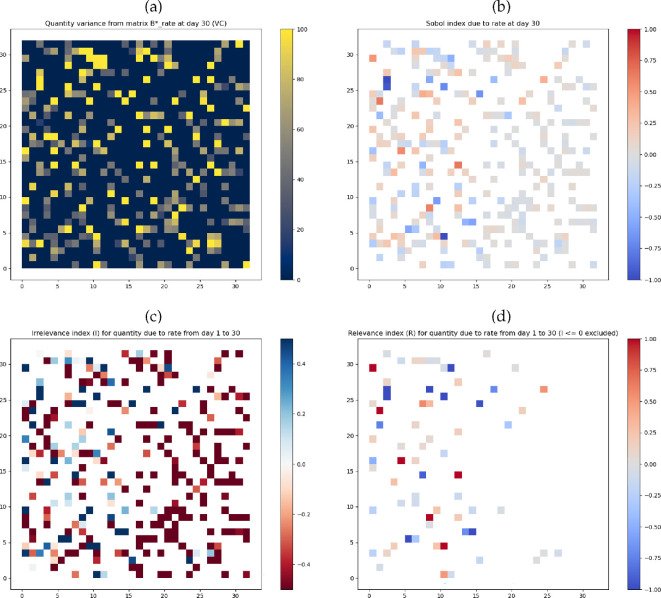
Smoking rates experiment ST-UA outcomes: (*a*) $VC_{rates|t=30}$ ; (*b*) total effects sensitivity index $U_{rates|t=30}$ ; (*c*) $I_{rates}$ where α=1 and β=1 ; and (*d*) $R_{rates}$ η=1 and ρ=0.5 .

The ST-UA outcomes of the experiments are shown in figures 3 and 4, and further visualizations are provided in the Appendix in electronic supplementary material, figures S3 and S4. We discuss the results in the following order for each experiment separately: (i) variances; (ii) total effects sensitivity indices; (iii) irrelevance indices; and (iv) relevance indices. We finally make comparisons between the experiments where relevant.

#### (i) Wages experiment

For the wages experiment, first, the variance maps (figure 3*a* and electronic supplementary material, figure S3a–d in the Appendix) highlight the areas occupied by human agents leading to cigarette purchases. The highest variance value in all the maps is approximately 100 cigarettes purchased on the respective day. Approximately 8.7% of the occupied spatial units reach a variance VC value of at least 90, and only 25% of these spatial units lie in the east. This indicates that the wider normal distribution of wages in the west contributes to higher observed variances. When comparing the variance maps, the differences are visually subtle; this is expected as they are generated from runs using relatively similar input matrices J (equations (4.2) and (4.3)).

Second, the total effects sensitivity indices in figure 3*b* visualize the $VC_{wages}$ values at day 30 (electronic supplementary material, figure S3-g and S3-f in the Appendix visualize the differences between the V and $VC_{wages}$ maps at days 1 and 30). For both days 1 and 30, the majority of the eastern areas have a sensitivity index close to zero, and only 4 (2%) of those eastern spatial units have a sensitivity index within the range of [−0.25, −0.1]. This means that, in these spatial units, the mean spatial purchases is more sensitive to inputs other than wages. This is expected as the narrow distribution of wages in the east leads to lower variability of wages at initialization, and accordingly a lower effect on the output. By contrast, the western side includes more spatial units having a sensitivity index within the range of [0.1, 0.25]; 9 spatial units in the west (6.6% of the western occupied areas) have a sensitivity index within that range at day 30. The mean spatial purchases in those units are more affected by the wages, which is caused by the wider normal distribution of wages at initialization in these areas. Comparing the sensitivity index maps at day 1 and day 30 to identify the propagation of the uncertainty index is visually challenging (see electronic supplementary material, figure S3-e and S3-f in the Appendix for reference). Further, the relatively low sensitivity indices within the wages experiment makes this a more demanding process. This is where the irrelevance and relevance indices are useful tools to highlight such propagation of the sensitivity index.

Third, the irrelevance index (figure 3*c*) is calculated using high α=2 and β=2 factors for $\left(\lambda_{i}=0.38\right)$ . This allows for representing a set of cases as relevant for analysis in this experiment, despite the low sensitivity indices ranging between −0.4 and 0.4 in all the spatial units. In the eastern area, the irrelevance index indicates that 131 spatial units (95.92% of the eastern spatial units) have a significantly low total effects sensitivity index at both day 1 and day 30. By contrast, only 6 spatial units in the east (4.37% of the eastern spatial units) have a significant sensitivity index at day 1, or day 2 or both. In the western areas, 84 spatial units (59.57% of western spatial units) are considered to have an insignificant sensitivity index at day 1 and day 30, and the rest have a significant sensitivity index at day 1, day 30 or both. These findings showcase that the wider statistical distribution of wages in the west leads to a high number of relevant spatial units for further uncertainty propagation analysis, and the narrower wages distribution in the east leads to a significantly lower number such relevant spatial units.

Finally, the uncertainty propagation relevance index (figure 3*d*) is calculated using high η=2 and ρ=1 values for a steeper curved function leading to an exaggeration of any minor propagation in the sensitivity index from day 1 to day 30. Assuming that the values highlighted as relevant are at $\lambda_{R}\approx 0.1$ , which corresponds to a $\lambda_{U}=0.37$ in table 3. Similar to the irrelevance index, this allows for emphasizing some cases in the wages experiment given the sensitivity index ranges from −0.4 to 0.4. The uncertainty propagation relevance index shows that the majority of the eastern spatial units have a value higher than zero. Five out of the 6 relevant spatial units in the east have a positive relevance index, with 4 having a relevance index above 0.75. In these spatial units, the mean spatial purchases are highly affected by the inputs at day 1, and they are highly affected by inputs other than wages at day 30. By contrast, the western areas are approximately equally divided between positive and negative propagation relevance index values. A total of 27 western spatial units (47.36% of the relevant spatial western units) have a negative uncertainty propagation relevance index, and 30 (52.63%) have a positive propagation index. This means that in all these relevant western spatial units, the mean spatial purchases are significantly affected by the inputs at day 1. At day 30, in approximately half of these spatial units the mean spatial purchases are affected by the input wages (positive relevance index), whereas in the other half the purchases are affected by inputs other than wages (negative relevance index).

#### (ii) Smoking rates experiment

For the smoking rates experiment, first, the variance maps (figure 4*a* and electronic supplementary material, figure S4-a–d in the Appendix) show a similar spatial structure to the wages experiment as this is kept consistent across all the runs. A high number of spatial units reach the value of 100 in each of the variance maps at day 1 or day 30. For instance, 75 spatial units (27.37% of the occupied spatial units) have a $VC_{rates}$ value of at least 90 at day 30. This is spatially distributed equally on both the east and west areas; 27.65% and 26.27% of the occupied eastern and western areas respectively have a variance $VC_{rates}$ exceeding 90 at day 30. By observing each variance map in isolation, this can lead to a conclusion that the different statistical distributions of smoking rates does not have a direct effect on the outcome. However, this does not imply that the uncertainty measure (which is a function of the variance) does not propagate/change from day 1 to day 30.

Second, the total sensitivity maps (figure 4*b* and electronic supplementary material, figure S4-e,f in the Appendix) show that the majority of the spatial units with values higher than 0.1 or lower than −0.1 lie in the western areas. To exemplify, 100 western spatial units (70.92% of the occupied western areas) fall within that range of values at day 30, compared with only 33 in the east (24.09% of the eastern areas). The number of spatial units with a significantly positive or negative total effects sensitivity index value is equally divided. In the western area at day 30, 20 (14.18%) of the spatial units have a sensitivity index value above 0.25 indicating the mean spatial purchases are strongly affected by the smoking rates. Similarly, 22 (15.6%) of the occupied western spatial units have a sensitivity index lower than 0.25 where inputs other than the smoking rates affect the mean spatial purchases. Overall, this shows a clear effect for the wider statistical distribution of the smoking rates in the western areas at both days 1 and 30. Comparing the maps of such days to identify the propagation of the sensitivity index is visually challenging similar to the wages experiments. However, the high values of the sensitivity index make it feasible in some cases (e.g. observe the eastern areas with a sensitivity index higher than 0.75 in electronic supplementary material, figure S4-e compared with figure S4-f in the Appendix 5).

Third, the irrelevance index (figure 4*c*) is calculated using the factors α=1 and β=1 for $\lambda_{i}=1$ representing no adjustments to the threshold of ignoring spatial units (see table 2). This is applied as the variance and total effects sensitivity indices widely range between −0.8 and 0.8. The majority of the eastern spatial units are recommended for exclusion from the analysis where 127 eastern spatial units (92.7% of the eastern spatial areas) have an irrelevance index below zero, compared with only 10 western spatial units (7.3%). This implies that the narrow distribution of smoking rates in the east leads to an insignificant sensitivity index at both day 1 and day 30 in the majority of the eastern areas. By contrast, the wider distribution of wages in the western areas contributes to a significant sensitivity index in at least one of days 1 and 30.

Finally, the uncertainty propagation relevance index (figure 4*d*) is calculated using the factors η=1 and ρ=0.5 . η=1 creates a flatter relevance index; a major propagation in the sensitivity index is required from day 1 to day 30 to affect the relevance index. ρ=0.5 minimizes the effect of edge cases with simultaneously high sensitivity index at day 1 and day 30. This is applicable in this experiment due to the relatively high values of the total effects sensitivity indices in the west at both day 1 and day 30—which are expected to yield significantly positive/negative relevance index values at low ρ . In the eastern areas, out of the 10 relevant spatial units, only 1 has a relevance index value lower than −0.5 and 1 has a value higher than 0.5. This indicates that, despite the narrow distributions of smoking rates in the east, the sensitivity index still highly propagates from day 1 to day 30 in some spatial units. These are areas of interest where further investigations of the effect of smoking rates and other inputs can improve the reliability of the outcomes and help further understand the spatial interactions in the model. In the western areas, a slightly higher number of spatial units have a significantly negative relevance index (11 spatial units) compared with the spatial units with a significantly positive relevance index (8 spatial units). This means that there is a relatively higher number of western spatial units where the mean spatial purchases are simultaneously affected by the inputs at day 1 and by inputs other than the smoking rate at day 30. This highlights areas where investigating inputs beyond the smoking rates is useful by reapplying the ST-UA approach using either the uncertainty due to the interaction of inputs with smoking rates (parameter and model uncertainty), or the sensitivity index for each other input.

#### (iii) Wages and smoking rates experiments: comparisons

Comparing both experiments, three key observations can be made. First, the variance in the smoking rates experiment is significantly higher than in the wages experiment across all the spatial units occupied by smokers (see figures 3*a* and 4*a*). This indicates that the mean spatial purchases are more sensitive to the smoking rates input than the wages input in the TT ABM. Second, the total effects sensitivity indices reach higher positive and lower negative values in the smoking rates experiment compared with the wages experiment (see figures 3*b* and 4*b*). This is a direct result of the higher variance in the smoking rates experiments. It further confirms that smoking rates have a more significant effect on the mean spatial purchases. Third, the irrelevance indices and the uncertainty propagation relevance index are visually similar across both experiments. This is because the factors ( α , β , η , ρ ) have been adjusted in each experiment to assure (i) the irrelevance thresholds do not include or exclude all the spatial units; and (ii) the steepness of the relevance index curves matches the major or minor changes in the propagation of the sensitivity index. This shows the flexibility of the ST-UA approach in terms of selecting the spatial units of relevance and visualizing the minor and major changes in temporal uncertainty propagation. In other words, it allows modellers to define how sensitive the visualizations are to the observed propagation of the selected uncertainty measure.

## 5. Conclusion

The spatial outcomes of models representing complex spatial systems are subject to inherent uncertainties due to the heterogeneous statistical distributions of inputs across the model space. Identifying and visualizing such spatial uncertainties is particularly relevant in cases where the models are used to directly inform spatial interventions or improve our understanding of the spatial dynamics of a given phenomenon. For this purpose, we developed a novel ST-UA approach to identify and visualize the temporal uncertainty propagation in spatial units due to specific model inputs. We showcased the ST-UA approach using a total effects uncertainty measure; a variance based index that can be calculated following a Monte Carlo sampling approach. We applied the approach on the TT ABM which simulates the smoking behaviour of individuals moving between home and work while having access to cigarette outlets [40]. We created two experiments that separately explore the impact of wages and smoking rates on the purchased cigarettes by smokers in each spatial unit. Each experiment divided the model space into western and eastern areas, and each used a wider normal distribution of either wages or smoking rates in the western areas. The selection of the input parameters has been deliberately designed to show two cases where the input has either a high impact (smoking rates) or low impact (wages) on the measured spatial outcome (mean number of purchased cigarette packs). The experiments highlighted that visually comparing multiple maps to identify uncertainty propagation is challenging, hence the need for using the relevance and irrelevance indicators of the ST-UA approach. These indicators showed clearly that wider distribution of a particular input leads to more instances of relevant uncertainty propagation—visually observed as a higher number of relevant spatial units in the western areas in both experiments (figures 3*d* and 4*d*). Comparing the maps across the experiments highlighted the flexibility of setting the factors in equations (3.1) and (3.2) to modify relevance and irrelevance index visualizations. For instance, a lower threshold of the irrelevance index $\lambda_{I}$ was used when considering the low total effects sensitivity measures in the wages experiment. Subsequently, a steeper relevance index curve was used yielding a lower $\lambda_{U}$ to visualize the minor changes in the sensitivity measure due to wages from day 1 to day 30.

Overall, the irrelevance and relevance indices of the ST-UA approach are useful tools for modellers to (i) communicate the spatio-temporal uncertainties of the models, particularly with non-experts in the field of behavioural simulations; and (ii) identify edge cases with high uncertainties, noting that the thresholds of the edge cases must be selected based on the uncertainty measure under analysis. In a realistic context, the developed models can be constructed in geospatial contexts, rather than abstract grids. This makes the visual maps further relatable to individuals that are familiar with the context under analysis. It also makes the boundaries of the areas where higher/lower uncertainty propagation observed more relatable (e.g. neighbourhoods or administrative areas where more information on smoking behaviours is necessary). This can assure spatial interventions are applied with prior knowledge of zones with high uncertainty propagation during the model runs.

In future research, we aim to build on the presented ST-UA approach in three key areas: (i) visualizing the temporal propagation of uncertainty given more than two time steps; (ii) providing a method for identifying the relevant time step at which the uncertainty highly propagates between a time step and its subsequent one—this requires (ii-a) visualizing a time series for the relevance index spatially at each time step in comparison with its prior time step and (ii-b) applying a clustering approach to find spatio-temporal zones where uncertainty highly propagates; (iii) showcasing the application of the ST-UA approach using an ABM with an explicit representation of a geospatial context; and (iv) applying the ST-UA approach using a different UA measure, for instance Shapley values.

## Data Availability

The paper uses an existing model and data available in Luke *et al*. [40]. The model and the analysis code are also available in the public repository https://github.com/YahyaGamal/ST-UA_TobaccoTown. Supplementary material is available online at [66].
